# Correction: Innovative snail-mucus-extract (SME)-coated nanoparticles exhibit anti-inflammatory and anti-proliferative effects for potential skin cancer prevention and treatment

**DOI:** 10.1039/d4ra90049a

**Published:** 2024-05-01

**Authors:** Valeria Consoli, Salvatore Petralia, Luca Vanella, Maria Gulisano, Ludovica Maugeri, Cristina Satriano, Lucia Montenegro, Angela Castellano, Valeria Sorrenti

**Affiliations:** a Department of Drug and Health Sciences, University of Catania Via Santa Sofia 64 95125 Catania Italy sorrenti@unict.it; b CERNUT-Research Centre for Nutraceuticals and Health Products, University of Catania 95125 Catania Italy; c CNR-Institute of Biomolecular Chemistry Via Paolo Gaifami 18 95126 Catania Italy; d NanoHybrid Biointerfaces Laboratory (NHBIL), Department of Chemical Sciences, University of Catania Viale Andrea Doria, 6 95125 Catania Italy; e Mediterranean Nutraceutical Extracts (Medinutrex) Via Vincenzo Giuffrida 202 95128 Catania Italy

## Abstract

Correction for ‘Innovative snail-mucus-extract (SME)-coated nanoparticles exhibit anti-inflammatory and anti-proliferative effects for potential skin cancer prevention and treatment’ by Valeria Consoli *et al.*, *RSC Adv.*, 2024, **14**, 7655–7663, https://doi.org/10.1039/D4RA00291A.

The authors regret that the author names were shown incorrectly in the original article. The corrected author list is as shown above.

The Royal Society of Chemistry apologises for these errors and any consequent inconvenience to authors and readers.

## Supplementary Material

